# Impact of Heavy Snowfall on Emergency Transport and Prognosis of Out-of-Hospital Cardiac Arrest Patients: A Nation-Wide Cohort Study

**DOI:** 10.1017/S1049023X23006040

**Published:** 2023-08

**Authors:** Kentaro Omatsu, Mieko Uchiyama, Utako Shimizu, Yiwei Ling, Shujiro Okuda, Yu Koyama

**Affiliations:** 1. Department of Nursing, Niigata University Graduate School of Health Sciences, Niigata, Japan; 2. Department of Emergency Medical Sciences, Niigata University of Health and Welfare, Niigata, Japan; 3. Bioinformatics Laboratory, Niigata University Graduate School of Medical and Dental Sciences, Niigata, Japan

**Keywords:** Emergency Medical Services response interval, heavy snowfall, neurologically-favorable outcomes, out-of-hospital cardiac arrest, survival

## Abstract

**Background:**

Out-of-hospital cardiac arrest (OHCA) is a significant global cause of mortality, and Emergency Medical Services (EMS) response interval is critical for survival and a neurologically-favorable outcome. Currently, it is unclear whether EMS response interval, neurologically-intact survival, and overall survival differ between snowy and non-snowy periods at heavy snowfall areas.

**Methods:**

A nation-wide population-based cohort of OHCA patients, registered from 2017 through 2019 in the All-Japan Utstein Registry, was divided into four groups according to areas (heavy snowfall area or other area) and seasons (winter or non-winter): heavy snowfall-winter, heavy snowfall-non-winter, other area-winter, and other area-non-winter. The first coprimary outcome was EMS response interval, and the secondary coprimary outcome was one-month survival and a neurologically-favorable outcome at one month.

**Results:**

A total of 337,781 OHCA patients were divided into four groups: heavy snowfall-winter (N = 15,627), heavy snowfall-non-winter (N = 97,441), other area-winter (N = 32,955), and other area-non-winter (N = 191,758). Longer EMS response intervals (>13 minutes) were most likely in the heavy snowfall-winter group (OR = 1.86; 95% CI, 1.76 to 1.97), and also more likely in heavy snowfall areas in non-winter (OR = 1.44; 95% CI, 1.38 to 1.50). One-month survival in winter was worse not only in the heavy snowfall area (OR = 0.86; 95% CI, 0.78 to 0.94) but also in other areas (OR = 0.91; 95% CI, 0.87 to 0.94). One-month neurologically-favorable outcomes were also comparable between heavy snowfall-winter and other area-non-winter groups.

**Conclusions:**

This study showed OHCA in heavy snowfall areas in winter resulted in longer EMS response intervals. However, heavy snowfall had little effect on one-month survival or neurologically-favorable outcome at one month.

## Introduction

Out-of-hospital cardiac arrest (OHCA) is a significant global cause of mortality and a pressing public health concern.^1–3^ Despite the improvement and implementation of various treatment strategies to improve survival from OHCA, there is still an inadequate favorable neurological prognosis for survivors after OHCA, and global survival rates are less than 10%.^3^ To enhance survival after OHCA, implementation of steps in the “chain of survival” concept^4^ are required, such as immediate recognition of cardiac arrest and activation of the Emergency Medical Services (EMS), early cardiopulmonary resuscitation (CPR), rapid defibrillation, and effective Advanced Life Support (ALS).^1,2,4^ Therefore, early EMS intervention is critical for survival from OHCA.

The EMS response interval, defined as the interval between receipt of an emergency call and the arrival of EMS on the scene, is a traditional performance measure for EMS systems in developed countries.^5,6^ The EMS response interval is also an important determinant of survival after OHCA.^7–9^ An upper limit of 13 minutes for EMS response interval, when bystanders provide defibrillation, is reportedly associated with improved one-month neurologically-intact survival.^7,8^ Therefore, an EMS response interval as short as possible is needed for a favorable neurologically-intact survival and overall survival. However, several factors can lengthen EMS response interval, such as traffic conditions, distance from the EMS station to the scene, an increased number of cases, and population density.^10–12^ In addition to these factors, weather conditions may affect EMS response interval due to disruptions or delays in the transportation network.

In general, so-called “bad weather conditions” often refer to heavy rain, strong wind, storm, or typhoon, but many of these are transient weather changes. On the other hand, there are seasonal weather conditions that produce changes in weather that persist over a certain period of time, especially low temperatures in the winter season. In fact, many Western countries, such as the United States and Europe, are hit by deep freezes several times during the winter season, which often cause disruptions in transportation networks and traffic jams. Snowfall is associated with an increased incidence of traffic disruptions^13^ and trauma injury.^14,15^ Heavy snowfall or a deep freeze may affect performance in components of the “chain of survival.” Worsened traffic conditions resulting from heavy snowfall may impede EMS response intervals and transport to hospital times, potentially leading to an adverse impact on outcomes following OHCA. In addition, there is reported to be an increased onset of OHCA during the winter season, and also an increase in poor outcomes associated with the winter season and cold environment.^16–18^ Moreover, an association between snowfall and onset of OHCA was reported from studies in the United States and Europe.^19,20^ Based on these findings, the risk of onset of OHCA increases in the winter season, while adverse weather conditions in snowy areas may prolong EMS response intervals and reduce neurologically-intact survival and overall survival.

So far, it is unclear whether EMS response interval, neurologically-intact survival, and overall survival differ between snowy and non-snowy periods at heavy snowfall areas, or whether there is a difference between heavy snowfall areas and non-heavy snowfall areas. The aims of the present study were to clarify whether there were any differences in EMS response interval and OHCA prognosis: (1) between heavy snowfall and non-heavy snowfall regions, and (2) between snowfall and non-snowfall seasons in heavy snowfall regions.

## Patients and Methods

### Study Design

This study was a retrospective, nation-wide population-based cohort study using data from the All-Japan Utstein Registry. The All-Japan Utstein Registry, maintained by the Fire and Disaster Management Agency (FDMA; Kasumigaseki, Chiyoda-ku, Tokyo, Japan), is a nation-wide population-based registry of OHCA that adheres to the standardized Utstein-style.^21,22^ The FDMA of Japan supervises the nation-wide EMS system, while local government fire departments operate the local EMS systems, which cover 99% of the population.^11^ Most EMS units contain at least one emergency life-saving technician (ELST), a highly trained individual who can practice some aspects of ALS. During the study period, all the EMS providers performed CPR and ALS according to the Japanese Resuscitation Council (Yoyogi, Shibuya-ku, Tokyo, Japan) guideline.^23^

### Data Collection

In Japan, patients with emergencies can make emergency calls without hesitation because ambulances are a part of administrative services and ambulance transport is free of charge. The study period was set from 2017 through 2019 to avoid the strain on EMS produced by the global coronavirus disease 2019 (COVID-19) pandemic in 2020, which may have impacted this research.

These OHCA data were obtained using a form that included patient information recommended by the Utstein-style reporting guidelines for cardiac arrest.^22^ In 2005, the FDMA initiated an on-going, nation-wide, population-based registry of all OHCA patients resuscitated by EMS. The data were collected by EMS personnel in collaboration with the OHCA patients’ treating physicians and submitted to the local fire department. The data were then integrated into the registry system on the FDMA database server. The data were checked for consistency by the database software program. If the data form was incomplete, the FDMA returned it to the local fire department and the data form was completed.^21^ The FDMA provided all the anonymous data to the study group. The database included the following data: event date and location (prefecture); sex; age; cause of OHCA; initial rhythm; bystander witness status, presence, and type of bystander CPR and/or public-access defibrillation (PAD) use; advanced airway management (AAM) and/or adrenaline administration by ELST; time of arrest; EMS call receipt; arrival time at the scene and CPR by EMS; prehospital return of spontaneous circulation; one-month survival; and neurological outcomes at one month after OHCA. The cause of OHCA was presumed to be of cardiac origin unless evidence suggested cerebrovascular disease, respiratory disease, malignant tumor, evident external cause, or other non-cardiac cause. The physicians in charge and EMS personnel determined the cause of the arrest. Neurological outcome was defined using the Cerebral Performance Category score.^22^ The EMS response interval was calculated as the duration from the call receipt by a dispatch center to ambulance arrival at the scene of the OHCA, as previously reported.^5–10^

This study included adult patients (age ≥18 years) with an episode of OHCA who received attempted resuscitation by EMS personnel in Japan from January 1, 2017 through December 31, 2019. The following patients were excluded: patients for whom cardiac arrests were witnessed by EMS personnel or for whom no resuscitation was attempted by EMS personnel, patients with inappropriate records of EMS responses (ie, EMS response interval <0 minutes or >120 minutes), and patients with transport time >180 minutes.

### Study Cohort

Japan, an island nation, is divided into 47 prefectures. Snowfall is prevalent during winter in Japan, except for some limited prefectures located in the south.^24^ Although there are some prefectures where partial heavy snowfall zones exist, ten prefectures have a heavy snowfall zone over the whole area. The Japanese Ministry of Land, Infrastructure, Transport, and Tourism (Kasumigaseki, Chiyoda-ku, Tokyo, Japan) has designated these ten prefectures as “heavy snowfall areas” (Supplementary Appendix, Figure S1; available online only).^14,24,25^ Therefore, the cohort of the present study was divided into four groups according to areas (heavy snowfall area or other area) and seasons (winter or non-winter). Winter was defined as December through February.

### Outcomes

The first coprimary outcome was EMS response interval, and a cutoff time of 13 minutes was used according to results from previous studies.^7,8^ The secondary coprimary outcome was one-month prognosis, indicated by both one-month survival and a one-month neurologically-favorable outcome. A neurologically-favorable outcome was defined as a Cerebral Performance Category score of CPC1 or CPC2.

### Statistical Analyses

Categorical variables are presented as counts (proportions), and continuous variables are presented as medians (interquartile range [IQR]). Baseline characteristics and study outcomes of categorical variables were compared using Chi-square tests and Kruskal-Wallis tests. Multivariate logistic regression analyses were performed to evaluate the association between groups and EMS response interval (<13 minutes). Potential confounders prior to EMS arrival in the analytical model were adjusted for the day of the week and the time of day when the OHCA occurred, based on data from previous studies.^26–28^ In addition, multivariate logistic regression analysis was performed to compare one-month outcomes by area-season. Potential prehospital confounders for the analytic model were selected based on biological plausibility and data from previous studies.^7,26–28^ The 12 selected variables included age, sex, witnessed arrest by bystander, some type of bystander CPR initiation, initial shockable rhythm, presumed cardiac cause, defibrillation by EMS, AAM by ELSTs, adrenaline administration by ELSTs, day of week (weekday as Monday-Friday), time of day (daytime as 9:00am-4:59pm), and area-season.

All statistical analyses were performed using JMP Pro 16.1.0 software (SAS Institute Inc.; Cary, North Carolina USA). The level of significance was set at P <.05.

### Ethics Approval

The FDMA and the institutional ethical review board of Niigata University (Niigata-City, Niigata, Japan) approved this study with a waiver of patient informed consent (#2021-0112).

## Results

### Patients

**Figure 1.**
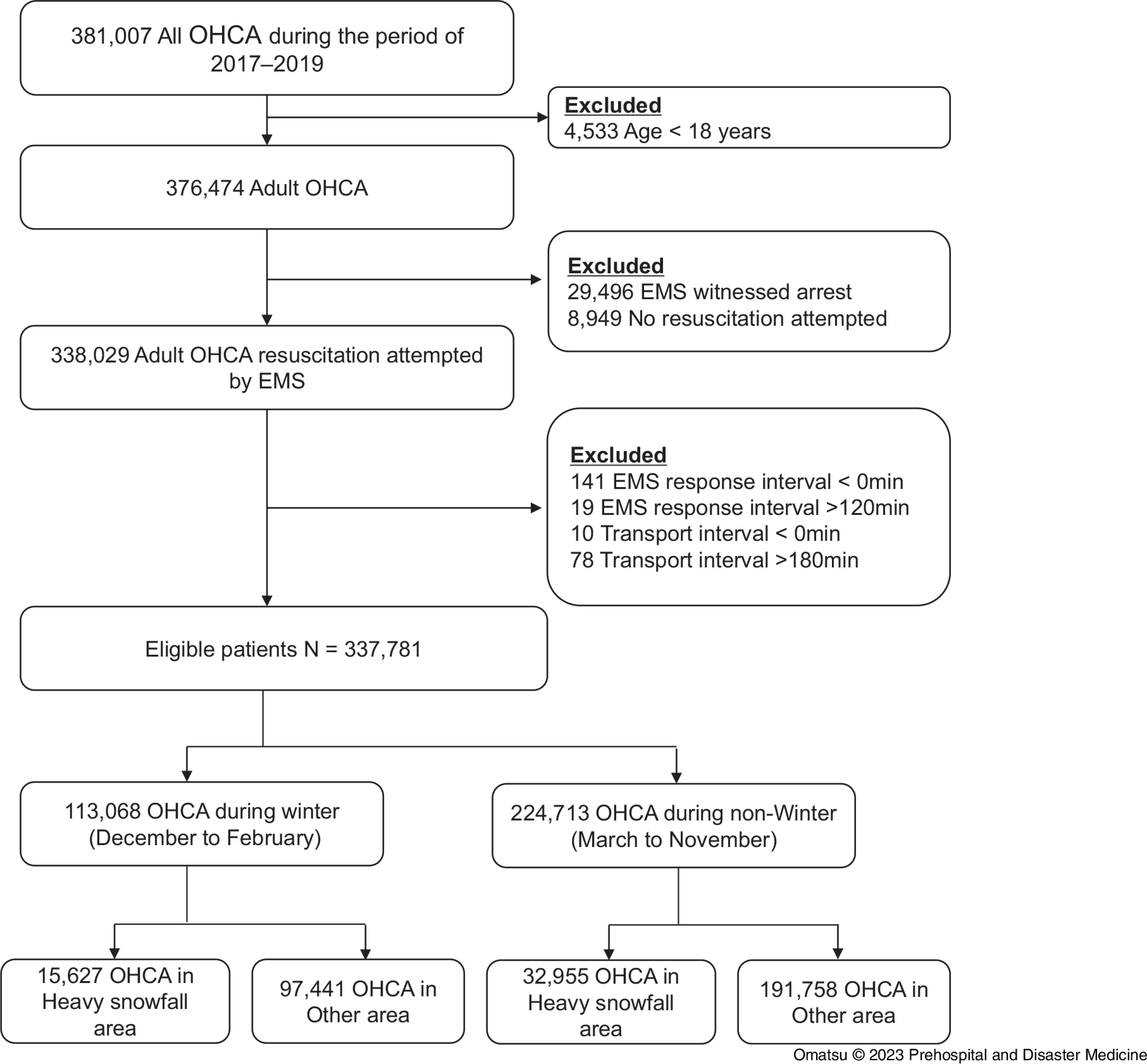
Flowchart of Patient Selection. Note: Patients aged 18 years or older with resuscitation attempted by EMS personnel after out-of-hospital cardiac arrest in Japan from 2017-2019 were included in this study. Abbreviations: OHCA, out-of-hospital cardiac arrest; EMS, Emergency Medical Services.

A total of 381,007 OHCA episodes were entered in the All-Japan Utstein Registry from 2017 through 2019, and 337,781 patients were eligible for enrollment in this study (Figure 1).

The study cohort was divided into four groups according to area (heavy snowfall or other area) and season (winter or non-winter): heavy snowfall-winter (N = 15,627), heavy snowfall-non-winter (N = 97,441), other area-winter (N = 32,955), and other area-non-winter (N = 191,758), as shown in Figure 1. The etiology of cardiac arrest was variable, with the most common cause presumed to be cardiac in all patients and in each group (Table 1; Supplementary Appendix, Table S1 - available online only).

**Table 1.** Baseline Characteristics of the Participants According to Season and Area

Characteristics	Total (N = 337,781)	Heavy Snowfall Area		Other Area		P Value
		Winter (N = 15,627)	Non-Winter (N = 32,955)	Winter (N = 97,441)	Non-Winter (N = 191,758)	
Year, n (%)^a^						<.001
2017	113,630 (33.6)	5,095 (32.6)	11,112 (33.7)	32,450 (33.3)	64,973 (33.9)	
2018	112,872 (33.4)	5,318 (34.0)	10,968 (33.3)	33,186 (34.1)	63,400 (33.1)	
2019	111,279 (32.9)	5,214 (33.4)	10,875 (33.0)	31,805 (32.6)	63,385 (33.1)	
Weekday, n (%)^a^	239,517 (70.9)	11,093 (71.0)	23,385 (71.0)	68,956 (70.8)	136,083 (71.0)	.72
Daytime, n (%)^a^	122,119 (36.2)	5,753 (36.8)	11,875 (36.0)	34,943 (35.9)	69,548 (36.3)	.049
Age in Years, median (IQR)	80 (69-87)	81 (71-88)	81 (69-88)	80 (70-87)	79 (68-87)	<.001
Male Sex, n (%)^a^	192,097 (56.9)	8,793 (56.3)	18,438 (56.0)	55,253 (56.7)	109,613 (57.2)	<.001
Cardiac Cause, n (%)^a^	212,196 (62.8)	9,705 (62.1)	19,467 (59.1)	62,716 (64.4)	120,308 (62.7)	<.001
Witnessed, n (%)^a^	121,135 (35.9)	5,078 (32.5)	11,328 (34.4)	32,854 (33.7)	71,875 (37.5)	<.001
Bystander CPR, n (%)^a^	181,463 (53.7)	8,727 (55.9)	18,563 (56.3)	51,029 (52.4)	103,144 (53.8)	<.001
PAD, n (%)^a^	4,819 (1.4)	156 (1.0)	445 (1.4)	1,136 (1.2)	3,082 (1.6)	<.001
Initial Shockable Rhythm, n (%)^a^	21,112 (6.3)	890 (5.7)	2,110 (6.4)	5,286 (5.4)	12,826 (6.7)	<.001
Defibrillation by EMS, n (%)^a^	31,837 (9.4)	1,525 (9.8)	3,124 (9.5)	8,593 (8.8)	18,595 (9.7)	<.001
Administration of Adrenaline by ELSTs, n (%)^a^	82,417 (24.4)	4,733 (30.3)	9,844 (29.9)	22,546 (23.1)	45,294 (23.6)	<.001
AAM by ELSTs, n (%)^a^	139,255 (41.2)	7,382 (47.2)	14,968 (45.4)	40,372 (41.4)	76,533 (39.9)	<.001
EMS Response Interval (min), Median (IQR)^b^	7 (6-9)	8 (6-10)	8 (6-10)	7 (6-9)	7 (6-9)	<.001
>13 minutes, n (%)^a^	21,865 (6.5)	1,650 (10.6)	2,750 (8.3)	6,033 (6.2)	11,432 (6.0)	<.001
EMS Transport Interval (min), Median (IQR)^b^	24 (19-31)	25 (19-33)	24 (19-31)	24 (19-31)	24 (19-31)	<.001
Prehospital ROSC, n (%)^a^	30,831 (9.1)	1,268 (8.1)	3,002 (9.1)	8,078 (8.3)	18,483 (9.6)	<.001
One-Month Survival, n (%)^a^	15,678 (4.6)	563 (3.6)	1,495 (4.5)	3,913 (4.0)	9,707 (5.1)	<.001
Neurologically-Favorable Outcome, n (%)^a^	7,662 (2.3)	245 (1.6)	731 (2.2)	1,826 (1.9)	4,860 (2.5)	<.001

Abbreviation: AAM, advanced airway management; CPR, cardiopulmonary resuscitation; ELST, emergency life-saving technician; EMS, Emergency Medical Services; PAD, public-access defibrillation; ROSC, return of spontaneous circulation.

a Categorical variables are presented as counts (proportions) and differences between groups were evaluated using the chi-square tests.

b Continuous variables are presented as medians (interquartile ranges [IQR]) and differences between groups were evaluated using the Kruskal–Wallis tests.

Cases of OHCA in winter occurred in 15,627 of 48,582 patients (32.2%) in heavy snowfall areas and 97,441 of 289,199 patients (33.7%) in other areas (Table 1; Supplementary Appendix, Table S3 - available online only). An EMS response interval of >13 minutes occurred in 4,400 of 48,582 patients (9.1%) in the heavy snowfall areas and in 17,465 of 289,199 patients (6.0%) in other areas (Table 1). The proportions of both one-month survival and a neurologically-favorable outcome were lowest in the heavy snowfall-winter group (563 of 15,627 patients [3.6%] and 245 of 15,627 patients [1.6 %], respectively) compared with other groups.

### EMS Response Interval

**Figure 2.**
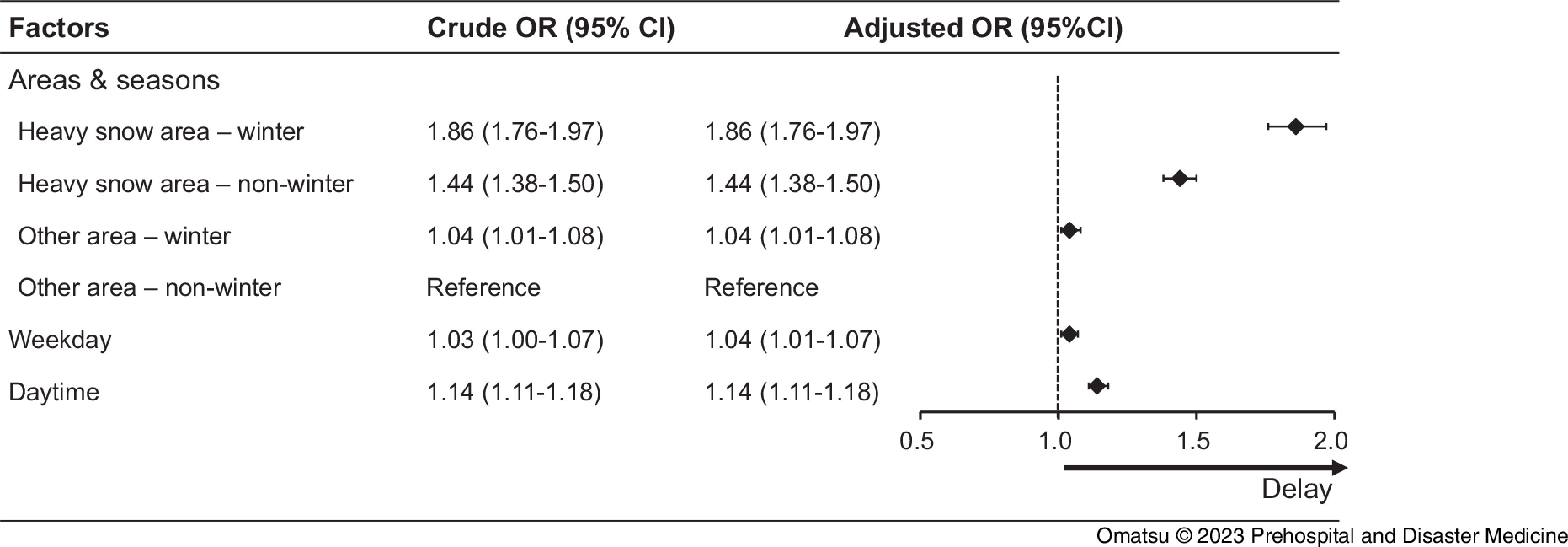
Crude and Adjusted ORs of Factors Associated with EMS Response Interval (>13 minutes). Abbreviations: OR, odds ratio; CI, confidence interval; EMS, Emergency Medical Services.

The EMS response interval was set as the first coprimary outcome among the four groups categorized according to areas and seasons. Table 1 and Figure 2 show that longer EMS response intervals (>13 minutes) were most likely to occur in the heavy snowfall-winter group (1,650 of 156,27 patients [10.6%]; OR = 1.86; 95% CI, 1.76 to 1.97); however, they were also likely to occur even in heavy snowfall areas in non-winter seasons (2,750 of 32,955 patients [8.3%]; OR = 1.44; 95% CI, 1.38 to 1.50). Cases of OHCA during daytime were also associated with a longer EMS response interval (OR = 1.14; 95% CI, 1.11 to 1.18).

### One-Month Prognosis

The secondary coprimary outcome in this study was the prognosis at one month, and two prognostic indicators were used: one-month survival and a neurologically-favorable outcome at one month.

One-month survival throughout the year in heavy snowfall areas and other areas was 4.2% (2,058 of 48,582 patients) and 4.7% (13,620 of 289,199 patients), respectively (Table 1; Supplementary Appendix, Table S7 - available online only). Survival at one month was more likely in cases of bystander-witnessed arrest (OR = 3.87; 95% CI, 3.72 to 4.02), bystander CPR (OR = 1.23; 95% CI, 1.19 to 1.27), initial shockable rhythm (OR = 4.17; 95% CI, 3.86 to 4.50), defibrillation by EMS (OR = 2.07; 95% CI, 1.92 to 2.22), or a daytime event (OR = 1.39; 95% CI, 1.34 to 1.44). On the other hand, one-month survival was significantly lower in cases of AAM by ELSTs (OR = 0.71; 95% CI, 0.69 to 0.74) or adrenaline administration by ELSTs (OR = 0.80; 95% CI, 0.77 to 0.83).

**Figure 3.**
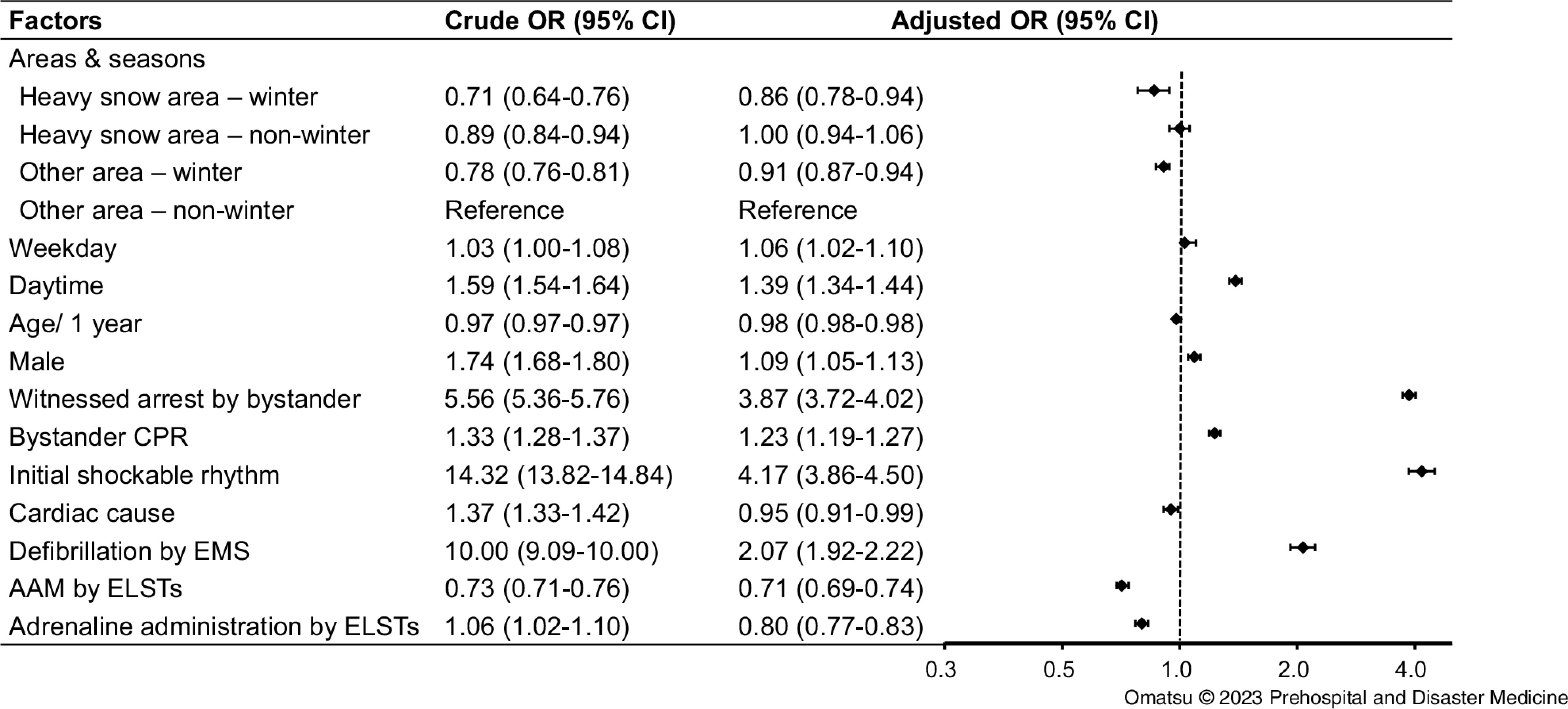
Crude and Adjusted ORs of Factors Associated with One-Month Survival. Abbreviations: OR, odds ratio; CI, confidence interval; CPR cardiopulmonary resuscitation; EMS, Emergency Medical Services; ELST, emergency life-saving technician; AAM, advanced airway management.

The impact of season showed one-month survival in winter was worse both in the heavy snowfall area (OR = 0.86; 95% CI, 0.78 to 0.94) and in other areas (OR = 0.91; 95% CI, 0.87 to 0.94). In the non-winter seasons, one-month survival was comparable between the heavy snowfall areas and other areas (Figure 3).

A neurologically-favorable outcome at one month after OHCA throughout the year in heavy snowfall areas and other areas was 2.0% (976 of 48,582 patients) and 2.3% (6,686 of 289,199 patients), respectively (Table 1; Supplementary Appendix, Table S8 - available online only). The neurologically-favorable outcome at one month was significantly more prevalent in males (OR = 1.21; 95% CI, 1.14 to 1.28), bystander-witnessed arrests (OR = 4.22; 95% CI, 3.98 to 4.48), bystander CPR (OR = 1.48; 95% CI, 1.40 to 1.56), initial shockable rhythm (OR = 4.32; 95% CI, 3.88 to 4.80), cardiac causes (OR = 1.60; 95% CI, 1.50 to 1.71), defibrillation by EMS (OR = 2.57; 95% CI, 2.31 to 2.86), or daytime events (OR = 1.40; 95% CI, 1. 33 to 1.47). Meanwhile, a one-month neurologically-favorable outcome was significantly less likely in cases of AAM by ELSTs (OR = 0.39; 95% CI, 0.37 to 0.42) or adrenaline administration by ELSTs (OR = 0.35; 95% CI, 0.32 to 0.37).

**Figure 4.**
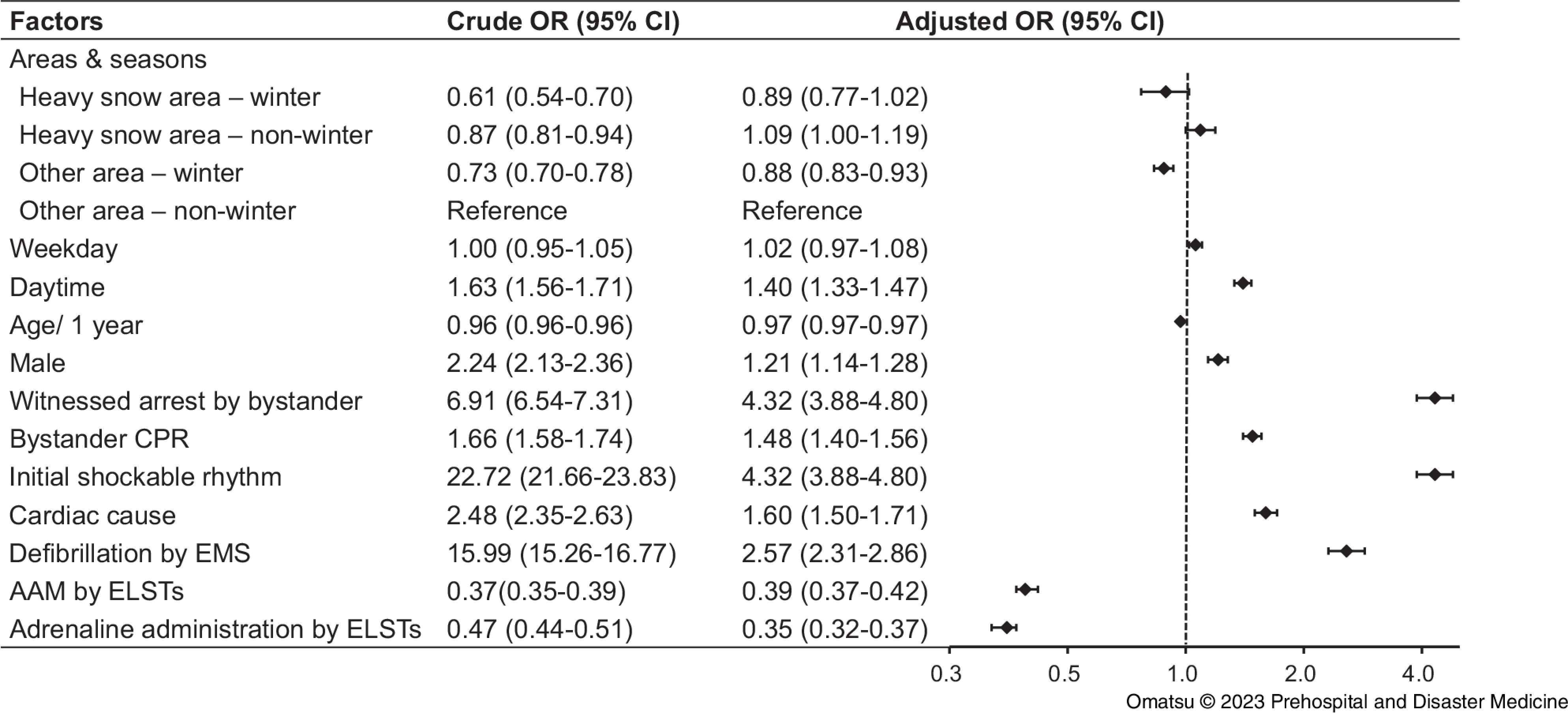
Crude and Adjusted ORs of Factors Associated with One-Month Neurologically-Favorable Outcome. Abbreviations: OR, odds ratio; CI, confidence interval; CPR cardiopulmonary resuscitation; EMS, Emergency Medical Services; ELST, emergency life-saving technician; AAM, advanced airway management.

The impact of area and season revealed neurologically-favorable outcomes at one month were comparable between the heavy snowfall-winter group and other area-non-winter group. In contrast, a neurologically-favorable outcome at one month was less likely in winter compared with non-winter in other areas (OR = 0.88; 95% CI, 0.83 to 0.93; Figure 4).

## Discussion

In this study using a nation-wide, population-based registry of OHCA, an independent association existed between OHCA in heavy snowfall areas in winter and delayed EMS response interval and decreased one-month survival, after adjusting for covariates. To the best of the authors’ knowledge, this is the first nation-wide study to evaluate whether longer EMS response intervals and lower survival results if OHCA occurs in a heavy snowfall area.

This survey period was set from 2017 through 2019, before the outbreak of the global COVID-19 pandemic, when emergency systems around the world, including Japan, were responding to an extraordinary situation. Since the COVID-19 pandemic is easing and societies are returning to the previous emergency situations, the emergency system that existed before the pandemic needs to be re-assessed. This target period was believed to be suitable for this study because the total number of OHCAs and the numbers in the four groups divided by area and season remained almost constant each year during the research period (Supplementary Appendix, Figure S2; available online only).

While a longer EMS response interval (>13 minutes) was significantly most likely to occur in heavy snowfall areas in winter (10.6%), it was also likely to occur even in the non-winter season (8.3%). There were more instances of EMS response intervals >13 minutes in the heavy snowfall area (9.1%) than in other areas (6.0%) throughout the year, suggesting some factors may lengthen EMS response intervals in heavy snowfall areas compared with other areas throughout the year. However, while the EMS response interval was not affected by the winter season in other areas, it is conceivable that the effect of winter on EMS response interval is stronger in the heavy snowfall area. The distribution of EMS centers in Japan is almost proportional to population density. Previous studies have shown that OHCA survival rates were lower in low-density areas due to longer EMS response intervals in these areas.^12^

In this study, one-month survival throughout the year in heavy snowfall areas and other areas was 4.2% and 4.7%, respectively (Table 1; Supplementary Appendix, Table S7). Meanwhile, one-month survival was decreased in both heavy snowfall areas and other areas in winter (Figure 3), and this finding might support the results of previous studies.^16–18^ The one-month survival rate reported here may be lower than reported from other countries.^3^ In Japan, almost all OHCA cases are emergency transported because EMS personnel are legally prohibited from terminating resuscitation without obviously dead cases: decapitation, incineration, decomposition, rigor mortis, or dependent cyanosis.^29^ Therefore, differences in the termination-of-resuscitation rule by country may influence the difference in survival rates.

A neurologically-favorable outcome at one month after OHCA throughout the year in heavy snowfall areas and other areas was 2.0% and 2.3%, respectively (Table 1; Supplementary Appendix, Table S8). This outcome might not be affected by the winter season as results were comparable between heavy snowfall-winter and other area-non-winter groups.

The rate of bystander CPR was slightly higher in heavy snowfall area either in winter or non-winter. In addition, the rate of ALS by ELSTs was higher in heavy snowfall areas. However, it is possible that these aggressive interventions were not performed in a timely manner. Thus, the execution of key elements of the “chain of survival” might be negatively affected if OHCA occurred in heavy snowfall area. Several studies have shown that a shorter EMS response interval is a critical determinant of survival after OHCA, and bystander CPR and PAD prolong the upper limit of EMS response interval.^8–10^

These findings suggest that several approaches are needed to reduce seasonal and regional differences in prolonged EMS response interval and outcomes after OHCA. First, residents need to understand that EMS response interval will be prolonged during the heavy snowfall periods and act to avoid increasing EMS demand. Second, strengthening of bystander CPR training may be effective for improving the survival of OHCA in the area with longer EMS response intervals. The development of more highly trained rescuers, such as “first-responder,” may also contribute to an improved OHCA survival rate.^30^ Third, the low PAD rates in heavy snowfall areas indicate the need for more effectively placed automated external defibrillators to improve OHCA outcomes.^21^

## Limitations

This study has some limitations. First, the categories for the All-Japan Utstein Registry data included the prefectures where OHCA occurred but not detailed location information. Second, the actual amount of snowfall was not measured because it was extremely difficult to obtain the exact information on the amount of snowfall at the point where OHCA occurred at that time. Therefore, the effects of snowfall outside of heavy snowfall areas could not be excluded. Third, these results were based on the Japanese health care system. In Japan, emergency calls and patient transport are free of charge. In countries where emergency calls and transportation fees are charged, hesitation to make emergency calls due to costs may also be a factor. However, these results will serve as a useful reference for countries with heavy snowfall in winter. Finally, as with all observational studies, data integrity validity and ascertainment bias are potentials.

## Conclusion

Cases of OHCA in heavy snowfall areas in winter resulted in longer EMS response intervals. However, heavy snowfall had little effect on one-month survival or neurologically-favorable outcome at one month.

## Supplementary material

For supplementary material accompanying this paper visit https://doi.org/10.1017/S1049023X23006040. click here to view supplementary material
