# Supplementary material for: Impact of Heavy Snowfall on Emergency Transport and Prognosis of Out-of-Hospital Cardiac Arrest Patients: A Nation-Wide Cohort Study
Source: Prehosp Disaster Med. 2023 Jul 14;38(4):436–43. doi: 10.1017/S1049023X23006040 (PMC10445114; doi:10.1017/S1049023X23006040)
Supplement: Supplementary file 1 [file S1049023X23006040sup001.docx]

**Supplementary Appendix**

**A study on the effect of heavy snowfall on the prognosis of out-of-hospital cardiac arrest patients.**

**Table of Contents**

**List of Study members**................................................................................... **3**

**Objectives**........................................................................................................ **4**

**Rationale**.......................................................................................................... **4**

**Clinical Hypothesis**......................................................................................... **4**

**Subject enrollment**.......................................................................................... **5**

**Analysis**............................................................................................................ **6**

**Supplementary Figures.**.................................................................................. **7**

**Supplementary Tables.**.................................................................................. **10**

**List of Study members**

Kentaro Omatsu^1^, M.Sc., Mieko Uchiyama^1^, Ph.D., Utako Shimizu^1^, Ph.D., Yiwei Ling^2^, Ph.D., Shujiro Okuda^2^, Ph.D., and Yu Koyama^1^, M.D., Ph.D.

Affiliations: ^1^Niigata University Graduate School of Health Science, Niigata, Japan, and ^2^ Bioinformatics Laboratory, Niigata University Graduate School of Medical and Dental Sciences, Niigata, Japan.

**1. OBJECTIVES**

**1.1 Primary**

The primary objective of this study was to clarify the impact of the weather environment, especially heavy snowfall, on the prognosis of out-of-hospital cardiac arrest (OHCA) patients.

**1.2 Secondary**

Secondary objective was contributing to the construction of a pre-hospital emergency medical system that matches the weather environment, especially heavy snowfall

**2. Rationale**

Out-of-hospital cardiac arrest (OHCA) is a significant global cause of mortality and a pressing public health concern, and emergency medical service (EMS) response time is an important determinant of survival after an OHCA. Snowfall is recognized to be associated with an increased incidence of traffic disruptions

**3. Clinical Hypothesis**

The prognosis of OHCA patients is particularly poor in heavy snowfall area compared to other area in winter.

There are some differences in EMS response time and the prognosis of OHCA between heavy snowfall area and non-heavy snowfall area.

**4. Subject enrollment**

**4.1 Data entry**

Data of OHCA were obtained using a form that included patients information recommended in the Utstein-style reporting guidelines for cardiac arrest. A total of 381,007 OHCA cases were registered into All-Japan Utstein Registry during 2017-2019, and finally, 337,781 patients were eligible for enrollment in this study.

**4.2 In- and exclusion criteria**

**Inclusion criteria**

- Adult patients (age ≥18 years) with an episode of OHCA who received attempted resuscitation by EMS personnel in Japan between January 1, 2017, and December 31, 2019.

**Exclusion criteria**

- Patients for whom cardiac arrests were witnessed by EMS personnel or for whom no resuscitation was attempted by EMS personnel
- Patients with inappropriate records on EMS responses as <0 min or >120 min
- Patients with transport time >180 min.

**4.3** **Study cohort**

Japan consists of 47 prefectures, and we define10 prefectures as heavy snowfall area according to the Japanese government (the Ministry of Land, Infrastructure, Transport and Tourism) designation, and we named other 37 prefectures as other area in this study. Winter is defined as December to February in this study.

The study cohort was divided into four groups according to areas (heavy snowfall or other areas) and seasons (winter or other seasons): heavy snowfall area-winter (N = 15,627), heavy snowfall area-non-winter (N = 97,441), other area-winter (N = 32,955) and other area-non-winter (N =1 91,758).

**5. Analysis**

**5.1** **Outcome setting**

6.1.1 The first coprimary outcome was EMS response time with the cutoff of 13 minutes is used according to results from previous studies [10,11].

6.1.2 The secondary coprimary outcome was

1) one-month survival

2) neurologically-favorable outcome at one-month, and the neurologically favorable outcome is defined as CPC score of 1 or 2.

**5.2** **Statistical analyses**

We used Kruskal-Wallis tests followed by Dunn post‐hoc test to analyze the continuous variables. Chi-square tests were performed for categorical variables. Multivariate logistic regression analyses were performed to evaluate the association between groups and EMS response time (<13 min). Potential confounders prior to EMS arrival in the analytical model were adjusted for the day of the week and the time of day when the OHCA occurred based on data from previous studies. In addition, multivariate logistic regression analysis was performed to compare one-month outcomes by areas-seasons. The potential prehospital confounders for the analytic model were selected based on biological plausibility and data from previous studies. The 12 selected variables included age, gender, witnessed arrest by bystander, some type of bystander CPR initiation, initial shockable rhythm, presumed cardiac cause, defibrillation by EMS, advanced airway management by ELSTs, adrenaline administration by ELSTs, day of week (weekday as Monday-Friday), time of day (daytime as 9:00-16:59) and areas - seasons.

All statistical analyses were performed using the JMP Pro 16.1.0 (SAS Institute Inc, Cary, NC). The level of significance was set at P < 0.05.

**Supplementary Figures**

**Figure S1. Geographic schema of classification of the study area. The Japanese government designated 10 of the 47 prefectures, primarily situated on the Sea of Japan side, as "heavy snowfall areas" for their entire prefectural area by an act on special measures for snow areas**.

**Figure S2. Onset of OHCA during 2017-2019.**

**Supplementary Tables**

| **Table S1. Etiology of cardiac arrest grouped by areas and seasons*** | | | | | | | |
| --- | --- | --- | --- | --- | --- | --- | --- |
|  |  | **Winter** | |  | **Non-winter** | |  |
| **Cause of OHCA** | **Overall**  **N = 337,781** | **Heavy Snowfall area**  **N = 15,627** | **Other area**  **N = 97,441** |  | **Heavy Snowfall area**  **N = 32,955** | **Other area**  **N = 191,758** | **P Value** |
| Presumed Cardiac origin | 212,196 (62.8) | 9,705 (62.1) | 62,716 (64.4) |  | 19,467 (59.1) | 120,308 (62.7) | <0.001 |
| Cerebrovascular disease | 9284 (2.7) | 471 (3.0) | 2,386 (2.4) |  | 1,027 (3.1) | 5,400 (2.8) |  |
| Choking | 26,208 (7.8) | 1,443 (9.2) | 7,149 (7.3) |  | 2,981 (9.0) | 14,635 (7.6) |  |
| Malignant tumor | 9,081 (2.7) | 378 (2.4) | 2,271 (2.3) |  | 910 (2.8) | 5,522 (2.9) |  |
| Exogenous | 19,691 (5.8) | 595 (3.8) | 4,564 (4.7) |  | 1,963 (6.0) | 12,569 (6.6) |  |
| Traffic injury | 5,423 (1.6) | 193 (1.2) | 1,296 (1.3) |  | 613 (1.9) | 3,321 (1.7) |  |
| Intoxication | 832 (0.2) | 42 (0.3) | 223 (0.2) |  | 99 (0.3) | 468 (0.2) |  |
| Drowning | 11,724 (3.5) | 701 (4.5) | 4,545 (4.7) |  | 1,295 (3.9) | 5,186 (2.7) |  |
| Hypothermia | 427 (0.1) | 67 (0.4) | 228 (0.2) |  | 43 (0.1) | 89 (0.05) |  |
| Anaphylaxis | 74 (0.02) | 1 (0.01) | 8 (0.01) |  | 12 (0.04) | 53 (0.03) |  |
| Others | 42,841 (12.7) | 2,031 (13.0) | 12,058 (12.4) |  | 4,545 (13.8) | 24,207 (12.6) |  |

* Categorical variables are presented as counts (proportions) and differences between groups were evaluated using the chi-square tests.

| **Table S2. Onset of OHCA: association between area and season*** | | | |
| --- | --- | --- | --- |
| **Areas** | **Winter** | **Non-winter** | **P Value** |
| Heavy snowfall area | 15,627 (32.2) | 32,955 (67.8) | P<0.001 |
| Other area | 97,441 (33.7) | 191,758 (66.3) |  |

* Categorical variables are presented as counts (proportions) and differences between groups were evaluated using the chi-square tests.

| **Table S3. Factors prior to EMS response time (>13 min) in all seasons*** | | |
| --- | --- | --- |
| **Factors** | **Adjusted OR (95% CI)**† | **P Value** |
| Areas & seasons |  |  |
| Heavy snowfall area | 1.55 (1.60 to 1.97) | <0.001 |
| Other area | Reference |  |
| Daytime | 1.14 (1.11 to 1.18) | <0.001 |
| Weekday | 1.14 (1.01 to 1.07) | 0.02 |

* Multivariate logistic regression analyses were performed.

† OR denotes odds ratio, CI confidence interval.

| **Table S4. Onset of OHCA: association between area and** **day of week*** | | | |
| --- | --- | --- | --- |
| **Areas** | **Weekday** | **Weekend** | **P Value** |
| Heavy snowfall area | 34478 (71.0) | 14104 (29.0) | 0.76 |
| Other area | 205039 (71.0) | 84160 (29.1) |  |

* Categorical variables are presented as counts (proportions) and differences between groups were evaluated using the chi-square tests.

| **Table S5. Onset of OHCA: association between and time zone*** | | | |
| --- | --- | --- | --- |
| **Areas** | **Daytime** | **Nighttime** | **P Value** |
| Heavy snowfall area | 17,628 (36.3) | 30,954 (63.7) | 0.52 |
| Other area | 104,491 (36.1) | 184,708 (63.9) |  |

* Categorical variables are presented as counts (proportions) and differences between groups were evaluated using the chi-square tests.

| **Table S6. Onset of OHCA: association between versus gender*** | | | |
| --- | --- | --- | --- |
| **Areas** | **Male** | **Female** | **P Value** |
| Heavy snowfall area | 27,231 (56.1) | 21,351 (44.0) | <0.001 |
| Other area | 164,866 (57.0) | 124,333 (43.0) |  |

* Categorical variables are presented as counts (proportions) and differences between groups were evaluated using the chi-square tests.

| **Table S7. Association between area and one-month survival*** | | | |
| --- | --- | --- | --- |
| **Areas** | **Survival** | **Death** | **P Value** |
| Heavy snowfall area | 2,058 (4.2) | 46,524 (95.8) | <0.001 |
| Other area | 13,620 (4.7) | 275,579 (95.3) |  |

* Categorical variables are presented as counts (proportions) and differences between groups were evaluated using the chi-square tests.

| **Table S8. Neurologically-favorable outcome at one-month*** | | | |
| --- | --- | --- | --- |
| **Areas** | **CPC 1-2** | **CPC 3-5** | **P Value** |
| Heavy snowfall area | 976 (2.0) | 47,606 (98.0) | <0.001 |
| Other area | 6,686 (2.3) | 282,513 (97.7) |  |

* Categorical variables are presented as counts (proportions) and differences between groups were evaluated using the chi-square tests.
